# Utilizing the LoxP-Stop-LoxP System to Control Transgenic ABC-Transporter Expression In Vitro

**DOI:** 10.3390/biom12050679

**Published:** 2022-05-08

**Authors:** Ikechukwu Esobi, Oladosu Olanrewaju, Jing Echesabal-Chen, Alexis Stamatikos

**Affiliations:** Department of Food, Nutrition, and Packaging Sciences, Clemson University, Clemson, SC 29634, USA; iesobi@g.clemson.edu (I.E.); oolados@g.clemson.edu (O.O.); jchen11@clemson.edu (J.E.-C.)

**Keywords:** metabolic regulation, reverse cholesterol transport, transduction

## Abstract

ABCA1 and ABCG1 are two ABC-transporters well-recognized to promote the efflux of cholesterol to apoAI and HDL, respectively. As these two ABC-transporters are critical to cholesterol metabolism, several studies have assessed the impact of ABCA1 and ABCG1 expression on cellular cholesterol homeostasis through ABC-transporter ablation or overexpressing ABCA1/ABCG1. However, for the latter, there are currently no well-established in vitro models to effectively induce long-term ABC-transporter expression in a variety of cultured cells. Therefore, we performed proof-of-principle in vitro studies to determine whether a LoxP-Stop-LoxP (LSL) system would provide Cre-inducible ABC-transporter expression. In our studies, we transfected HEK293 cells and the HEK293-derived cell line 293-Cre cells with ABCA1-LSL and ABCG1-LSL-based plasmids. Our results showed that while the ABCA1/ABCG1 protein expression was absent in the transfected HEK293 cells, the ABCA1 and ABCG1 protein expression was detected in the 293-Cre cells transfected with ABCA1-LSL and ABCG1-LSL, respectively. When we measured cholesterol efflux in transfected 293-Cre cells, we observed an enhanced apoAI-mediated cholesterol efflux in 293-Cre cells overexpressing ABCA1, and an HDL2-mediated cholesterol efflux in 293-Cre cells constitutively expressing ABCG1. We also observed an appreciable increase in HDL3-mediated cholesterol efflux in ABCA1-overexpressing 293-Cre cells, which suggests that ABCA1 is capable of effluxing cholesterol to small HDL particles. Our proof-of-concept experiments demonstrate that the LSL-system can be used to effectively regulate ABC-transporter expression in vitro, which, in turn, allows ABCA1/ABCG1-overexpression to be extensively studied at the cellular level.

## 1. Introduction

Normal cholesterol homeostasis is critical for human health [1]. Dysregulations in cholesterol homeostasis are associated with numerous conditions, which include cardiovascular disease, neurodegenerative disorders, and certain types of cancer [2]. Removing excess cholesterol from cells is imperative to cholesterol homeostasis, as cells are unable to catabolize cholesterol effectively [3,4]. Furthermore, toxicity may also occur from high intracellular cholesterol levels [5].

The removal of cellular cholesterol is called cholesterol efflux and there are four pathways in humans that regulate this process [6]. The two active cholesterol efflux pathways are regulated by two transporters known as ABCA1 and ABCG1 [6]. It has been traditionally recognized that ABCA1 exclusively effluxes cholesterol to circulatory apoAI, while ABCG1 effluxes cholesterol to circulatory HDL [7]. However, evidence has emerged to indicate that ABCA1 can also efficiently efflux cholesterol to smaller HDL particles, too [8].

Both ABCA1 and ABCG1 are generally considered to be atheroprotective due to their ability to remove excess cholesterol from peripheral tissues [9]. Surprisingly though, studies involving the whole-body and tissue-specific deletion and overexpression of ABCA1 and ABCG1 have been conflicting when evaluating whether these two transporters protect against atherosclerosis [10,11,12,13,14,15,16,17,18,19,20,21,22,23,24,25,26,27,28,29]. Therefore, it is possible that genetic manipulation of ABCA1/ABCG1 in certain cells or tissues at different stages of life may influence atherogenesis or atherosclerosis progression differently [30]. Studies that have focused on overexpressing ABCA1 and ABCG1 systemically or in certain cells/tissues have been conducted using approaches involving transgenesis and adenoviral-mediated somatic gene transfer [15,16,19,21,22,23,24]. However, these two strategies have limitations that include an inability to control overexpression at a specific point in time for the former [31,32], and transient overexpression for the latter [33]. There are also issues with determining the role ABCA1 and ABCG1 have at the cellular level, as there are no well-established in vitro models that involve inducing long-term ABCA1/ABCG1 overexpression in cultured cells at a desired time point. Understanding how ABCA1 and ABCG1 modulate cholesterol metabolism in numerous cell types, at various stages, that are being exposed to a variety of stimuli, can aid scientists in identifying cell-specific atheroprotective roles of ABCA1/ABCG1 during diverse conditions.

The LoxP-Stop-LoxP (LSL) system is a method to control transgenic expression at a defined time point. Within this system, transgene expression should only be initiated in the presence of the enzyme Cre recombinase [34], and Cre is able to excise the LoxP sites [35] that flank the stop sequence, allowing transgene expression to be initiated. Therefore, this system is a promising strategy for controlling ABCA1 and ABCG1 transgene expression in cultured cells. In this study, we investigated whether the LSL-system could be utilized to control ABCA1/ABCG1 transgenic expression in vitro. In our experiments, we used HEK293 and 293-Cre cells, which are two cell lines that do not normally express ABCA1/ABCG1 [36,37,38,39]. 293-Cre cells are similar to HEK293 cells, but have been modified to express Cre recombinase [37]. When transfecting these cell lines with plasmids containing ABCA1-LSL and/or ABCG1-LSL cassettes, robust ABCA1 and ABCG1 protein expression was detected in the 293-Cre cells, as determined by immunoblotting. To determine whether these ABC-transporters were functional in the 293-Cre transfected cells, we measured cholesterol efflux in 293-Cre cells overexpressing either ABCA1 and/or ABCG1 and showed that cholesterol efflux was enhanced in these cells when compared to control 293-Cre cells that did not have ABC-transporter transgene expression. Our results show that the LSL-system appears to be a powerful approach for controlling transgenic ABC-transporter expression in cultured cells and could be implemented when in vitro ABCA1/ABCG1 expression needs to be activated at a specific time point.

## 2. Materials and Methods

### 2.1. Cell Culture Maintenance

HEK293 cells were purchased from American Type Culture Collection (Manassas, VA, USA) and 293-Cre cells were provided as a kind gift from Dr. Frank Graham [37]. HEK293 cells were cultured in standard growth medium containing high-glucose DMEM (Corning, New York, NY, USA), 10% FB Essence (VWR Life Science Seradigm, Radnor, PA, USA), and 1% Pen-Strep (Corning). The standard growth medium used to maintain 293-Cre cells was similar to what was used for the HEK293 cells, with the only exception being the addition of G418 (500 μg/mL; VWR Life Science Seradigm) to the standard growth medium used for culturing 293-Cre cells. Medium for both cell types was replenished every 48–72 h. For cell maintenance, both HEK293 cells and 293-Cre cells were incubated within 10 cm cell culture plates at 37 °C under 5% CO_2_ conditions, and the confluency of cultured cells was based on coverage [40].

### 2.2. Plasmid Transfection of Cultured Cells

Using standard growth medium, both HEK293 cells and 293-Cre cells were grown in 6-well cell culture plates for immunoblotting experiments, and 293-Cre cells only were grown in 48-well cell culture plates for cholesterol efflux studies. Once cells reached 70–80% confluency, cells either remained untreated, vehicle-treated, or transfected with 2 μg of total plasmid for the immunoblotting experiments as follows: (1) empty vector plasmid; (2) ABCA1-LSL plasmid and empty vector plasmid; (3) ABCG1-LSL plasmid and empty vector plasmid; (4) ABCA1-LSL plasmid and ABCG1-LSL plasmid. For cholesterol efflux assays, only transfected 293-Cre cells were used for these experiments. When the 293-Cre cells being used for cholesterol efflux studies reached 70–80% confluency, they were transfected with 250 ng of total plasmid as described above for the immunoblotting experiments. As the cells used for cholesterol efflux assays were plated in 48-well plates, a lower number of cells were used per well when compared to the immunoblotting experiments where 6-well plates were used, and so less plasmid DNA was used to transfect the cells for the cholesterol efflux assays. All plasmids used in our studies were constructed by VectorBuilder (Chicago, IL, USA) and contained the ubiquitous CAG promoter [41] that permits constitutive transgene expression [42]. The empty vector plasmid used was a CAG promoter-only control (i.e., no transgene), while the LSL-based plasmids employed contained three repeated SV40 pA sequences flanked by two LoxP sites, allowing the LSL sequence to be excised by the enzyme Cre recombinase [43]. This LSL sequence within these plasmids is flanked by the CAG promoter and transgene, which allows Cre-induced transgene expression [34,43]. For cells that were transfected with two plasmids in our experiments, an equal mass of plasmid was used for these respective transfections. To prevent unequal plasmid DNA mass being used for transfections, cultured cells transfected with either ABCA1-LSL plasmid or ABCG1-LSL plasmid alone were also transfected with the empty vector plasmid to keep the plasmid mass identical among all groups. For the immunoblotting experiments, cells were either vehicle-treated or transfected using jetOPTIMUS transfection reagent (Polyplus, New York, NY, USA) for 24 h, 36 h, 48 h, 60 h, or 72 h. The reason we transfected cultured cells for up to 72 h was to determine whether transgenic ABCA1/ABCG1 protein expression may still be induced in 293-Cre cells, but absent in HEK293 cells. For the cholesterol efflux assays, plasmid DNA was transfected into 293-Cre cells using jetOPTIMUS transfection reagent for 6 h.

### 2.3. Western Blotting

Immunoblotting procedures were conducted as previously described [44]. Post-transfections, medium was removed, cells were washed with PBS, and protein was harvested from cells by using the RIPA lysis buffer and mammalian protease inhibitors (VWR Life Science). A BCA assay (BioVision, Milpitas, CA, USA) was used for measuring protein concentrations from the harvested cell lysates. We used SDS–PAGE to separate equal amounts of proteins within the cell lysate samples and then transferred the separated proteins onto PVDF membranes (Merck Millipore Ltd., Burlington, MA, USA). The molecular weight ladder used for immunoblotting was ExcelBand™ All Blue Broad Range Protein Marker (SMOBIO Technology, Hsinchu City, Taiwan/ROC). We blocked PVDF membranes with blocking buffer [44] and then probed for ABCA1 (1:1000 dilution, sc-58219; Santa Cruz Biotechnology, Dallas, TX, USA), ABCG1 (1:5,000 dilution, NB400-132; Novus Biologicals, Littleton, CO, USA), and loading control, GAPDH (1:1,000 dilution, sc-365062; Santa Cruz Biotechnology, Dallas, TX, USA). The secondary antibodies used included HRP-conjugated goat anti-rabbit IgG (1:10,000 dilution, HAF008; Novus Biologicals) and HRP-conjugated goat anti-mouse IgG (1:10,000 dilution, AP181P; Sigma-Aldrich, St. Louis, MO, USA). Bound secondary antibodies were detected with ECL substrate (Immobilon ECL Ultra Western HRP Substrate; MilliporeSigma, Billerica, MA, USA). Post-ECL incubation, we used a ChemiDoc system (Analytik Jena US, Upland, CA, USA) to perform imaging analysis.

### 2.4. Cholesterol Efflux Assays

Cholesterol efflux assays were performed as previously described [45,46]. Briefly, after transfecting the 293-Cre cells for 6 h, we removed standard growth medium containing the transfection reagent and untransfected plasmid DNA, washed cells with PBS, and then cultured cells in serum-free DMEM containing 2 mg/mL of fatty acid-free bovine serum albumin (Sigma-Aldrich), 1% pen-strep, and [^3^H] cholesterol (1 μCi/mL; PerkinElmer, Waltham, MA, USA) for 18 h, to cholesterol-load cells. To measure apoAI/HDL-mediated cholesterol efflux in 293-Cre cells, we first removed the medium used for cholesterol-loading, washed cells with PBS, and then incubated cultured cells with cholesterol efflux medium that contained either vehicle only, 5 μg/mL of apoAI, or 10 μg/mL of either HDL2, HDL3, or heterogenous HDL particles (Academy Bio-Medical Company, Houston, TX, USA), which contain both HDL2 and HDL3 [47], for 24 h. This experimental design utilized allowed cultured cells to efflux cholesterol to the cholesterol acceptors 24 h after initiating transfection, and cholesterol efflux was measured for a total of 24 h. Therefore, cholesterol efflux was measured in cultured cells 24–48 h post-transfection. Protein concentrations of the cholesterol acceptors used were determined by the Lowry method. The cholesterol efflux medium we used was identical in composition to the cholesterol-loading medium, minus the [^3^H] cholesterol. After treating cells with either vehicle only or the cholesterol acceptors, we filtered the medium to remove nonadherent cells, washed 293-Cre cells with PBS, and lysed cells as previously described [45,46]. Using a liquid scintillation counter (LS 6500; Beckman Coulter, Brea, CA, USA), we counted [^3^H] in the cells/medium, and then calculated apoAI/HDL-mediated cholesterol efflux as previously described [45,46].

## 3. Results

### 3.1. The LSL-System Is Effective in Restricting Transgenic ABC-Transporter Expression

**Figure 1:**
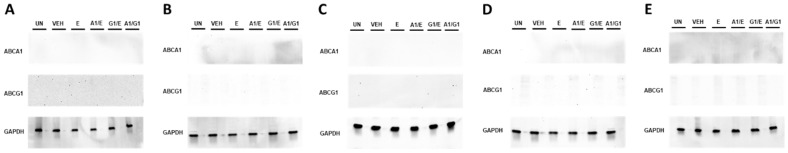
ABC-transporter protein expression is absent in HEK293 cells transfected with ABCA1-LSL and ABCG1-LSL-based plasmids. (**A**–**E**) Cultured HEK293 cells were either untreated (UN), vehicle-treated (VEH), transfected with empty vector plasmid (E), transfected with ABCA1-LSL plasmid and empty vector plasmid (A1/E), transfected with ABCG1-LSL plasmid and empty vector plasmid (G1/E), or transfected with ABCA1-LSL and ABCG1-LSL plasmids (A1/G1). Post-treatments, cell lysates were harvested 24 h (**A**), 36 h (**B**), 48 h (**C**), 60 h (**D**), or 72 h (**E**) after the respective treatments. (**A**–**E**) Western blotting was used for attempted detection of ABCA1 and ABCG1 proteins in cell lysates, with GAPDH used as a loading control.

We initially attempted to detect the presence of ABC-transporter expression in HEK293 cells that were either untreated, vehicle-treated for 24–72 h, or transfected with the following plasmids for 24–72 h: (1) empty vector; (2) ABCA1-LSL and empty vector; (3) ABCG1-LSL and empty vector; (4) ABCA1-LSL and ABCG1-LSL. We used HEK293 cells to determine whether ABC-transporter expression is prevented by the LSL-system, as HEK293 cells are an easily transfectable cell line that do not normally express ABCA1 or ABCG1 [36,38,39]. For our results, we failed to observe ABC-transporter expression in any of the HEK293 cells exposed to the untreated, vehicle-treated, and plasmid-transfected conditions (Figure 1A–E). These results confirm that the LSL-system effectively inhibits ABCA1 and ABCG1 transgene expression when Cre recombinase is not present.

### 3.2. Cre Recombinase Effectively Induces Transgene Expression of ABCA1-LSL and ABCG1-LSL

**Figure 2:**
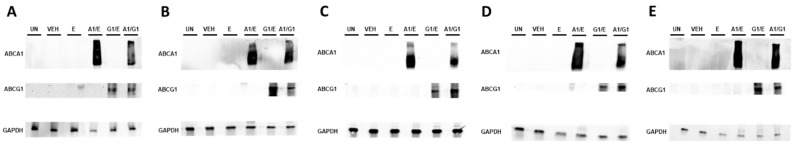
Robust ABCA1/ABCG1 protein expression is detected in 293-Cre cells transfected with ABCA1-LSL and ABCG1-LSL-based plasmids. (**A**–**E**) Cultured 293-Cre cells were either untreated (UN), vehicle-treated (VEH), transfected with empty vector plasmid (E), transfected with ABCA1-LSL plasmid and empty vector plasmid (A1/E), transfected with ABCG1-LSL plasmid and empty vector plasmid (G1/E), or transfected with ABCA1-LSL and ABCG1-LSL plasmids (A1/G1). Post-treatments, cell lysates were harvested 24 h (**A**), 36 h (**B**), 48 h (**C**), 60 h (**D**), or 72 h (**E**) after the respective treatments. (**A**–**E**) Immunoblotting was used to assess ABCA1, ABCG1, and GAPDH (loading control) protein expression in cell lysates.

293-Cre cells are considered an identical cell line to HEK293 cells, with an exception being that the 293-Cre cell line stably expresses Cre recombinase [37]. For these reasons, we used 293-Cre cells to introduce Cre into our in vitro system to directly test whether Cre recombinase can initiate transgenic ABC-transporter expression in cultured cells transfected with ABCA1-LSL and/or ABCG1-LSL-based plasmids. To test this, we used an experimental design similar to the sets of experiments outlined in Figure 1, with the difference being that 293-Cre cells were utilized instead of HEK293 cells. Our results showed robust protein expression of ABCA1 or ABCG1 only being detected in 293-Cre cells that were transfected with the ABCA1-LSL and/or ABCG1-LSL-based plasmids, respectively (Figure 2A–E). These results imply that ABCA1/ABCG1 transgene expression can be controlled within in vitro models by employing an LSL-system that only initiates expression when Cre is introduced to the cultured cells.

### 3.3. Cre-Induced Transient Expression of ABCA1 and ABCG1 Transgenes Enhances Cholesterol Efflux in 293-Cre Cells

**Figure 3:**
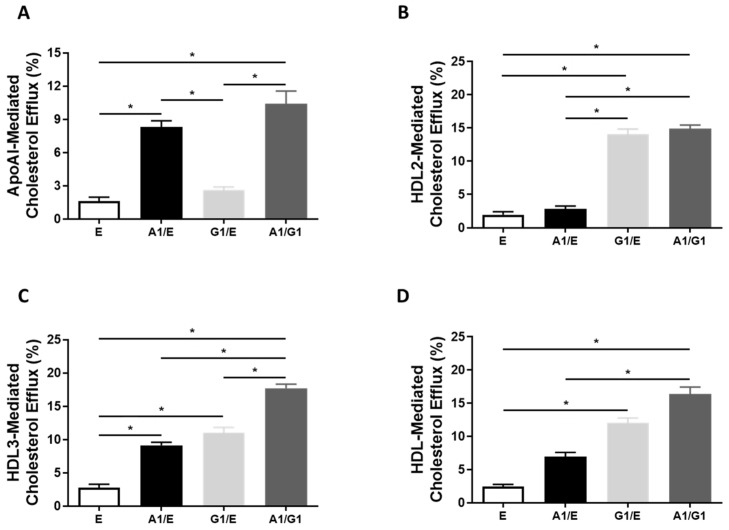
Enhanced cholesterol acceptor-mediated efflux in cultured 293-Cre cells constitutively expressing ABCA1 and ABCG1. (**A**–**D**) Cholesterol efflux measured in [^3^H] cholesterol-loaded 293-Cre cells transfected with either empty vector plasmid (E), ABCA1-LSL plasmid and empty vector plasmid (A1/E), ABCG1-LSL plasmid and empty vector plasmid (G1/E), or ABCA1-LSL and ABCG1-LSL plasmids (A1/G1), and incubated with either apoAI (**A**), HDL2 (**B**), HDL3 (**C**), or heterogenous HDL (**D**) particles. Three independent experiments with three biological replicates per treatment for each experiment. Data are mean ± SEM. Asterisks (*) indicate statistical significance (*p* < 0.05).

To test whether transfecting 293-Cre cells with ABCA1-LSL and/or ABCG1-LSL-based plasmids causes an increase in cholesterol acceptor-mediated cholesterol efflux in this cell line, we measured apoAI, heterogeneous HDL, HDL2, (i.e., large HDL particles), and HDL3 (i.e., small HDL particles)-mediated cholesterol efflux in 293-Cre cells transfected with the following plasmids: (1) empty vector; (2) ABCA1-LSL and empty vector; (3) ABCG1-LSL and empty vector; (4) ABCA1-LSL and ABCG1-LSL. As expected, apoAI-mediated cholesterol efflux was only enhanced in 293-Cre cells expressing ABCA1, and HDL2-mediated cholesterol efflux was only increased in 293-Cre cells expressing ABCG1 (Figure 3A,B). However, HDL3-mediated cholesterol efflux was shown to be increased in 293-Cre cells expressing both ABCG1 and ABCA1 alone, with an additive effect in cholesterol efflux being observed in the 293-Cre cells co-expressing transgenic ABCA1/ABCG1 (Figure 3C). Moreover, an increase in heterogenous HDL-mediated cholesterol efflux was observed in 293-Cre cells expressing ABCA1 and ABCG1 simultaneously when compared to control 293-Cre cells and 293-Cre cells expressing ABCA1 alone, but not in 293-Cre cells expressing ABCG1 alone. Additionally, while enhanced (heterogenous) HDL-mediated cholesterol efflux was observed in 293-Cre cells expressing ABCG1 alone when compared to 293-Cre control cells, no significant difference was observed when these ABCG1-overexpressing 293-Cre cells were compared to the 293-Cre cells expressing ABCA1 alone (Figure 3D). These results demonstrate that ABCA1 is capable of efficiently effluxing cholesterol to smaller HDL particles, which has been previously demonstrated [8,48].

## 4. Discussion

In this study, we wanted to demonstrate that employing an in vitro LSL-system is an effective way to control ABCA1 and ABCG1 transgene expression in cultured cells through the Cre-activated transgenic expression of ABCA1/ABCG1. When we transfected HEK293 cells and the HEK293-derived cell line 293-Cre cells with ABCA1-LSL and/or ABCG1-LSL-based plasmids, we only detected the robust expression of transgenic ABCA1/ABCG1 in the Cre-expressing 293-Cre cells. Thus, the in vitro model used in our experiments shows proof-of-concept that an LSL-system can be successfully utilized when the transgenic ABC-transporter expression needs to be induced at a precise point in time. From these results, we propose that the LSL-system may be utilized to overexpress ABC-transporters at a desired time point under specific stimuli. However, we do acknowledge that a limitation to our sets of experiments was not measuring ABCA1/ABCG1 transgenic expression under a variety of stimuli and diverse conditions.

In our experiments, we also measured cholesterol acceptor-mediated cholesterol efflux in the transfected 293-Cre cells, and as expected, increased apoAI-mediated cholesterol efflux was observed in ABCA1-overexpressing 293-Cre cells and increased HDL2-mediated cholesterol efflux was observed in 293-Cre cells overexpressing ABCG1. An appreciable amount of cholesterol being effluxed to small HDL particles was also observed in ABCA1-overexpressing 293-Cre cells, which was shown in the heterogenous HDL- and HDL3-mediated cholesterol efflux assays, indicating that small HDL particles can participate in ABCA1-mediated cholesterol efflux. While our data strongly suggest ABC-transporter functionality, including a catalytically deficient ABCA1/ABCG1 mutant as a negative control might further strengthen our findings, as this may be considered to be a superior negative control over empty vector plasmids [49]. Furthermore, incorporating appropriate, well-established ABCA1/ABCG1 positive/negative controls into future respective immunoblots may enhance experimental observations, and so should be strongly considered for future studies [8,11,50]. Additionally, we also acknowledge that analyzing cellular localization of the transgenic ABC-transporters and assessing function using other novel approaches would strengthen our cholesterol efflux assay results, as these proposed experiments are sometimes performed when assessing both overexpression and function [51]. Moreover, we recognize from our immunoblot experiments that a lack of replication and including only one experiment per condition may be viewed as a weakness within our study.

ABCA1 and ABCG1 function has been analyzed in various tissues/organs by using ABCA1*^fl/fl^*:ABCG1*^fl/fl^* mice and tissue-specific Cre-driver mice [11,25,26,52,53,54,55]. When using inducible Cre mouse lines, ABC-transporter expression can also be ablated at a certain point in time [56]. Moreover, culturing cells from ABCA1*^fl/fl^*:ABCG1*^fl/fl^* floxed mice ex vivo and then transducing these cells with a Cre-expressing viral vector permits some control [57,58] over when ABC-transporter expression would be ablated from these cultured cells. In addition, developing immortalized cell lines from these floxed ABCA1/ABCG1 cells would provide even greater flexibility on when ABC-transporter expression can be ablated, as this would eliminate the issues of primary cells exhibiting a short lifespan in culture [59,60]. However, with regard to inducing ABCA1- and/or ABCG1-overexpression at a precise time in vivo, there are currently no animal models generated that display this ability (to the authors’ knowledge), which prevents the possibility of employing ex vivo approaches to induce ABCA1/ABCG1-overexpression in cultured cells, as described above.

Viral vectors are a potential option to constitutively express ABC-transporters in vivo, as viral vectors that overexpress ABCA1 or ABCG1 can be injected intravenously into mice at various stages of life. However, there are challenges when using viral vectors to overexpress ABC-transporters in vivo. For instance, if ABCA1/ABCG1-overexpression needs to be restricted to a certain tissue or cell type, a tissue-specific promoter would need to be used along with a very high transduction efficiency to occur in the cells of interest. While lentivirus transduces a broad range of cells and can provide stable transgene expression through genome integration, lentivirus has a packaging capacity that can become problematic [61,62] for accommodating an expression cassette containing an ABCA1 transgene. This in turn may result in inefficient lentiviral production and sub-optimal transduction efficiency when attempting to use an ABCA1-overexpressing lentiviral vector [62]. Moreover, intravenous lentiviral injections have been shown to poorly transduce large arteries [63], which makes this route of administration ineffective for overexpressing ABC-transporters within the vessel wall through the use of viral vectors [33]. Though adenoviral vectors can effectively package larger transgenes such as ABCA1 [64] (and ABCG1 [19]), tropism is a concern when using adenovirus, as adenoviral vectors are unable to transduce certain cells efficiently [65,66,67]. Another concern with adenoviral vectors is that they only provide transient transgene expression due to adenoviral vector genomes remaining episomal and so loss of transgene expression occurs during repeated cell division [68,69,70]. 

Some of the challenges described above for viral vector-mediated ABCA1/ABCG1-overexpression in vivo are also present when using viral vectors to overexpress ABC-transporters in vitro, which hinders the ability for assessing any potential therapeutic role robust, long-term ABCA1, and/or ABCG1 expression might exhibit at the cellular level. Furthermore, as plasmid transfection is poor in certain cultured cells and transgene expression from plasmid transfection is transient [71,72], this unfortunately cannot be used as an alternative to test for any possible long-term therapeutic effects of ABCA1/ABCG1-overexpression at the cellular level. To overcome these concerns, stable cell lines can be generated [73] via incorporating ABCA1-LSL and/or ABCG1-LSL into their respective genomes. As we demonstrate in our proof-of-concept experiments involving transfected HEK293 and 293-Cre cells, ABC-transporter expression would not be initiated in these newly developed stable cell lines until the cells are exposed to Cre. Introducing Cre recombinase to the stable cell lines can be easily accomplished via transfecting cultured cells with a Cre-expressing plasmid for easily transfectable cell lines. For difficult-to-transfect cells, transducing cultured cells with either a Cre-expressing adenoviral vector, lentiviral vector, or AAV vector can be performed instead. Exposing Cre-expressing plasmids or viral vectors to the newly developed cell lines should result in robust and permanent [43] ABC-transporter transgene expression within the cultured cells that have been successfully transfected or transduced. As producing stable cell lines is relatively simple, fast, and economical [60,73], this method could easily be employed to determine what therapeutic role(s) robust ABC-transporter expression has at the cellular level, and allows scientists to initiate long-term ABCA1/ABCG1 transgene expression at any specific time, regardless of what experimental conditions are being used in vitro.

Data from our cholesterol efflux assays provided us with some expected and intriguing results, when cholesterol efflux was measured in transfected 293-Cre cells exposed to various cholesterol acceptors. While we anticipated increased apoAI-mediated cholesterol efflux in 293-Cre cells constitutively expressing ABCA1 and enhanced HDL2-mediated cholesterol efflux in ABCG1-overexpressing 293-Cre cells, we also observed a substantial amount of both heterogenous HDL- and HDL3-mediated cholesterol efflux in the ABCA1-overexpressing 293-Cre cells. However, the considerable amount of cholesterol efflux observed in the ABCA1-overexpressing 293-Cre cells exposed to small HDL particles accords with data that demonstrates ABCA1 is capable of efficiently effluxing cholesterol to smaller HDL particles [8], and also suggests that ABCA1 is the primary contributor to total cholesterol efflux via participating in both apoAI and small HDL-mediated cholesterol efflux [48], while ABCG1 plays a minor, more supportive role in cellular cholesterol efflux. Another interesting point to mention in our cholesterol efflux data is that we observed trivial amounts of cholesterol efflux in HDL-mediated cholesterol efflux in 293-Cre cells not expressing ABCA1 and ABCG1. While we initially assumed we would detect higher levels of HDL-mediated cholesterol efflux in this group, it is possible that the low levels of HDL-mediated cholesterol efflux observed in the ABCA1/ABCG1 non-expressing 293-Cre cells is from the 293-Cre cells being a HEK293-derived cell line, and HEK293 cells only express negligible amounts of SR-BI [74]. As SR-BI is able to participate in HDL-mediated cholesterol efflux [6], this may partially explain why we observed such low levels of HDL-mediated cholesterol efflux in control 293-Cre cells not expressing ABCA1 and ABCG1.

In conclusion, we demonstrate proof-of-concept how implementing an in vitro LSL-system can effectively control ABC-transporter expression in cultured cells through Cre-mediated activation. Utilizing in vitro models that employ this LSL-system to regulate constitutive ABCA1 and/or ABCG1 expression is superior to overexpressing transgenic ABCA1/ABCG1 expression in cultured cells via plasmid transfection or transduction using viral vectors. Lastly, when robust ABCA1 expression occurred in 293-Cre cells transfected with an ABCA1-LSL plasmid, we observed an appreciable increase in smaller HDL particle-mediated cholesterol efflux in addition to apoAI-mediated cholesterol efflux. These results suggest that ABCA1 appears to participate in both apoAI- and (small) HDL-mediated cholesterol efflux, which would imply that ABCA1 plays a predominant role in total cholesterol efflux. Hence, ABCA1-dependent cholesterol efflux may better facilitate the removal of excess cholesterol when compared to ABCG1, with ABCA1 contributing to the efflux of cellular cholesterol by interacting with multiple cholesterol acceptors.

## Data Availability

The data that support the findings of this study are available from the corresponding author upon reasonable request.

## References

[1] Goedeke L., Fernandez-Hernando C. (2012). Regulation of cholesterol homeostasis. Cell Mol. Life Sci..

[2] Luo J., Yang H., Song B.L. (2020). Mechanisms and regulation of cholesterol homeostasis. Nat. Rev. Mol. Cell Biol..

[3] Huang L.H., Elvington A., Randolph G.J. (2015). The role of the lymphatic system in cholesterol transport. Front. Pharmacol..

[4] Jesch E.D., Carr T.P. (2017). Food Ingredients That Inhibit Cholesterol Absorption. Prev. Nutr. Food Sci..

[5] Song Y., Liu J., Zhao K., Gao L., Zhao J. (2021). Cholesterol-induced toxicity: An integrated view of the role of cholesterol in multiple diseases. Cell Metab..

[6] Phillips M.C. (2014). Molecular mechanisms of cellular cholesterol efflux. J. Biol. Chem..

[7] Yvan-Charvet L., Wang N., Tall A.R. (2010). Role of HDL, ABCA1, and ABCG1 transporters in cholesterol efflux and immune responses. Arter. Thromb. Vasc. Biol..

[8] Du X.M., Kim M.J., Hou L., Le Goff W., Chapman M.J., Van Eck M., Curtiss L.K., Burnett J.R., Cartland S.P., Quinn C.M. (2015). HDL particle size is a critical determinant of ABCA1-mediated macrophage cellular cholesterol export. Circ. Res..

[9] Frambach S., de Haas R., Smeitink J.A.M., Rongen G.A., Russel F.G.M., Schirris T.J.J. (2020). Brothers in Arms: ABCA1- and ABCG1-Mediated Cholesterol Efflux as Promising Targets in Cardiovascular Disease Treatment. Pharmacol. Rev..

[10] Yvan-Charvet L., Ranalletta M., Wang N., Han S., Terasaka N., Li R., Welch C., Tall A.R. (2007). Combined deficiency of ABCA1 and ABCG1 promotes foam cell accumulation and accelerates atherosclerosis in mice. J. Clin. Investig..

[11] Westerterp M., Tsuchiya K., Tattersall I.W., Fotakis P., Bochem A.E., Molusky M.M., Ntonga V., Abramowicz S., Parks J.S., Welch C.L. (2016). Deficiency of ATP-Binding Cassette Transporters A1 and G1 in Endothelial Cells Accelerates Atherosclerosis in Mice. Arter. Thromb. Vasc. Biol..

[12] Wang X., Collins H.L., Ranalletta M., Fuki I.V., Billheimer J.T., Rothblat G.H., Tall A.R., Rader D.J. (2007). Macrophage ABCA1 and ABCG1, but not SR-BI, promote macrophage reverse cholesterol transport in vivo. J. Clin. Investig..

[13] Van Eck M., Singaraja R.R., Ye D., Hildebrand R.B., James E.R., Hayden M.R., Van Berkel T.J. (2006). Macrophage ATP-binding cassette transporter A1 overexpression inhibits atherosclerotic lesion progression in low-density lipoprotein receptor knockout mice. Arter. Thromb. Vasc. Biol..

[14] Van Eck M., Bos I.S., Kaminski W.E., Orso E., Rothe G., Twisk J., Bottcher A., Van Amersfoort E.S., Christiansen-Weber T.A., Fung-Leung W.P. (2002). Leukocyte ABCA1 controls susceptibility to atherosclerosis and macrophage recruitment into tissues. Proc. Natl. Acad. Sci. USA.

[15] Vaisman B.L., Demosky S.J., Stonik J.A., Ghias M., Knapper C.L., Sampson M.L., Dai C., Levine S.J., Remaley A.T. (2012). Endothelial expression of human ABCA1 in mice increases plasma HDL cholesterol and reduces diet-induced atherosclerosis. J. Lipid Res..

[16] Singaraja R.R., Fievet C., Castro G., James E.R., Hennuyer N., Clee S.M., Bissada N., Choy J.C., Fruchart J.C., McManus B.M. (2002). Increased ABCA1 activity protects against atherosclerosis. J. Clin. Investig..

[17] Out R., Hoekstra M., Hildebrand R.B., Kruit J.K., Meurs I., Li Z., Kuipers F., Van Berkel T.J., Van Eck M. (2006). Macrophage ABCG1 deletion disrupts lipid homeostasis in alveolar macrophages and moderately influences atherosclerotic lesion development in LDL receptor-deficient mice. Arter. Thromb. Vasc. Biol..

[18] Out R., Hoekstra M., Habets K., Meurs I., de Waard V., Hildebrand R.B., Wang Y., Chimini G., Kuiper J., Van Berkel T.J. (2008). Combined deletion of macrophage ABCA1 and ABCG1 leads to massive lipid accumulation in tissue macrophages and distinct atherosclerosis at relatively low plasma cholesterol levels. Arter. Thromb. Vasc. Biol..

[19] Munch G., Bultmann A., Li Z., Holthoff H.P., Ullrich J., Wagner S., Ungerer M. (2012). Overexpression of ABCG1 protein attenuates arteriosclerosis and endothelial dysfunction in atherosclerotic rabbits. Heart. Int..

[20] Lu H. (2015). Daugherty A: Atherosclerosis. Arter. Thromb. Vasc. Biol..

[21] Kennedy M.A., Barrera G.C., Nakamura K., Baldan A., Tarr P., Fishbein M.C., Frank J., Francone O.L., Edwards P.A. (2005). ABCG1 has a critical role in mediating cholesterol efflux to HDL and preventing cellular lipid accumulation. Cell Metab..

[22] Joyce C.W., Wagner E.M., Basso F., Amar M.J., Freeman L.A., Shamburek R.D., Knapper C.L., Syed J., Wu J., Vaisman B.L. (2006). ABCA1 overexpression in the liver of LDLr-KO mice leads to accumulation of pro-atherogenic lipoproteins and enhanced atherosclerosis. J. Biol. Chem..

[23] Feng Y., Lievens J., Jacobs F., Hoekstra M., Van Craeyveld E., Gordts S.C., Snoeys J., De Geest B. (2010). Hepatocyte-specific ABCA1 transfer increases HDL cholesterol but impairs HDL function and accelerates atherosclerosis. Cardiovasc. Res..

[24] Brunham L.R., Singaraja R.R., Duong M., Timmins J.M., Fievet C., Bissada N., Kang M.H., Samra A., Fruchart J.C., McManus B. (2009). Tissue-specific roles of ABCA1 influence susceptibility to atherosclerosis. Arter. Thromb. Vasc. Biol..

[25] Bi X., Zhu X., Gao C., Shewale S., Cao Q., Liu M., Boudyguina E., Gebre A.K., Wilson M.D., Brown A.L. (2014). Myeloid cell-specific ATP-binding cassette transporter A1 deletion has minimal impact on atherogenesis in atherogenic diet-fed low-density lipoprotein receptor knockout mice. Arter. Thromb. Vasc. Biol..

[26] Bi X., Zhu X., Duong M., Boudyguina E.Y., Wilson M.D., Gebre A.K., Parks J.S. (2013). Liver ABCA1 deletion in LDLrKO mice does not impair macrophage reverse cholesterol transport or exacerbate atherogenesis. Arter. Thromb. Vasc. Biol..

[27] Attie A.D., Hamon Y., Brooks-Wilson A.R., Gray-Keller M.P., MacDonald M.L., Rigot V., Tebon A., Zhang L.H., Mulligan J.D., Singaraja R.R. (2002). Identification and functional analysis of a naturally occurring E89K mutation in the ABCA1 gene of the WHAM chicken. J. Lipid Res..

[28] Aiello R.J., Brees D., Francone O.L. (2003). ABCA1-deficient mice: Insights into the role of monocyte lipid efflux in HDL formation and inflammation. Arter. Thromb. Vasc. Biol..

[29] Aiello R.J., Brees D., Bourassa P.A., Royer L., Lindsey S., Coskran T., Haghpassand M., Francone O.L. (2002). Increased atherosclerosis in hyperlipidemic mice with inactivation of ABCA1 in macrophages. Arter. Thromb. Vasc. Biol..

[30] Van Eck M., Van Berkel T.J. (2013). ATP-binding cassette transporter A1 in lipoprotein metabolism and atherosclerosis: A new piece of the complex puzzle. Arter. Thromb. Vasc. Biol..

[31] Saunders T.L. (2020). The History of Transgenesis. Methods Mol. Biol..

[32] Murphy D., Carter D.A. (1993). Introduction to transgenesis. Methods Mol. Biol..

[33] Lee C.S., Bishop E.S., Zhang R., Yu X., Farina E.M., Yan S., Zhao C., Zheng Z., Shu Y., Wu X. (2017). Adenovirus-Mediated Gene Delivery: Potential Applications for Gene and Cell-Based Therapies in the New Era of Personalized Medicine. Genes Dis..

[34] Sharma S., Zhu J. (2014). Immunologic applications of conditional gene modification technology in the mouse. Curr. Protoc. Immunol..

[35] Kim H., Kim M., Im S.K., Fang S. (2018). Mouse Cre-LoxP system: General principles to determine tissue-specific roles of target genes. Lab. Anim. Res..

[36] Toth K., Wold W.S. (2002). HEK? No!. Mol. Ther..

[37] Chen L., Anton M., Graham F.L. (1996). Production and characterization of human 293 cell lines expressing the site-specific recombinase Cre. Somat. Cell Mol. Genet..

[38] Marsche G., Frank S., Raynes J.G., Kozarsky K.F., Sattler W., Malle E. (2007). The lipidation status of acute-phase protein serum amyloid A determines cholesterol mobilization via scavenger receptor class B, type I. Biochem. J..

[39] Kobayashi A., Takanezawa Y., Hirata T., Shimizu Y., Misasa K., Kioka N., Arai H., Ueda K., Matsuo M. (2006). Efflux of sphingomyelin, cholesterol, and phosphatidylcholine by ABCG1. J. Lipid Res..

[40] Segeritz C.-P., Vallier L., Jalali M., Saldanha F.Y.L., Jalali M. (2017). Chapter 9—Cell Culture: Growing Cells as Model Systems In Vitro. Basic Science Methods for Clinical Researchers.

[41] Niwa H., Yamamura K., Miyazaki J. (1991). Efficient selection for high-expression transfectants with a novel eukaryotic vector. Gene.

[42] Fukuda T., Mishina Y., Walker M.P., Di Augustine R.P. (2005). Conditional transgenic system for mouse aurora a kinase: Degradation by the ubiquitin proteasome pathway controls the level of the transgenic protein. Mol. Cell Biol..

[43] Bapst A.M., Dahl S.L., Knopfel T., Wenger R.H. (2020). Cre-mediated, loxP independent sequential recombination of a tripartite transcriptional stop cassette allows for partial read-through transcription. Biochim. Biophys. Acta Gene Regul. Mech..

[44] Huang K., Jo H., Echesabal-Chen J., Stamatikos A. (2021). Combined LXR and RXR Agonist Therapy Increases ABCA1 Protein Expression and Enhances ApoAI-Mediated Cholesterol Efflux in Cultured Endothelial Cells. Metabolites.

[45] Stamatikos A., Knight E., Vojtech L., Bi L., Wacker B.K., Tang C., Dichek D.A. (2020). Exosome-Mediated Transfer of Anti-miR-33a-5p from Transduced Endothelial Cells Enhances Macrophage and Vascular Smooth Muscle Cell Cholesterol Efflux. Hum. Gene Ther..

[46] Esobi I.C., Barksdale C., Heard-Tate C., Powell R.R., Bruce T.F., Stamatikos A. (2021). MOVAS Cells: A Versatile Cell Line for Studying Vascular Smooth Muscle Cell Cholesterol Metabolism. Lipids.

[47] Eren E., Yilmaz N., Aydin O. (2012). High Density Lipoprotein and it’s Dysfunction. Open Biochem. J..

[48] Heinecke J.W. (2015). Small HDL promotes cholesterol efflux by the ABCA1 pathway in macrophages: Implications for therapies targeted to HDL. Circ. Res..

[49] Prelich G. (2012). Gene overexpression: Uses, mechanisms, and interpretation. Genetics.

[50] Westerterp M., Murphy A.J., Wang M., Pagler T.A., Vengrenyuk Y., Kappus M.S., Gorman D.J., Nagareddy P.R., Zhu X., Abramowicz S. (2013). Deficiency of ATP-binding cassette transporters A1 and G1 in macrophages increases inflammation and accelerates atherosclerosis in mice. Circ. Res..

[51] Deprey K., Batistatou N., Kritzer J.A. (2020). A critical analysis of methods used to investigate the cellular uptake and subcellular localization of RNA therapeutics. Nucleic Acids Res..

[52] Soliman E., Bhalla S., Elhassanny A.E.M., Malur A., Ogburn D., Leffler N., Malur A.G., Thomassen M.J. (2022). Myeloid ABCG1 Deficiency Enhances Apoptosis and Initiates Efferocytosis in Bronchoalveolar Lavage Cells of Murine Multi-Walled Carbon Nanotube-Induced Granuloma Model. Int. J. Mol. Sci..

[53] Timmins J.M., Lee J.Y., Boudyguina E., Kluckman K.D., Brunham L.R., Mulya A., Gebre A.K., Coutinho J.M., Colvin P.L., Smith T.L. (2005). Targeted inactivation of hepatic Abca1 causes profound hypoalphalipoproteinemia and kidney hypercatabolism of apoA-I. J. Clin. Investig..

[54] Chung S., Timmins J.M., Duong M., Degirolamo C., Rong S., Sawyer J.K., Singaraja R.R., Hayden M.R., Maeda N., Rudel L.L. (2010). Targeted deletion of hepatocyte ABCA1 leads to very low density lipoprotein triglyceride overproduction and low density lipoprotein hypercatabolism. J. Biol. Chem..

[55] Zhu X., Lee J.Y., Timmins J.M., Brown J.M., Boudyguina E., Mulya A., Gebre A.K., Willingham M.C., Hiltbold E.M., Mishra N. (2008). Increased cellular free cholesterol in macrophage-specific Abca1 knock-out mice enhances pro-inflammatory response of macrophages. J. Biol. Chem..

[56] Feil S., Valtcheva N., Feil R. (2009). Inducible Cre mice. Methods Mol. Biol..

[57] Han S.H., Malaga-Dieguez L., Chinga F., Kang H.M., Tao J., Reidy K., Susztak K. (2016). Deletion of Lkb1 in Renal Tubular Epithelial Cells Leads to CKD by Altering Metabolism. J. Am. Soc. Nephrol..

[58] Hu J.H., Wei H., Jaffe M., Airhart N., Du L., Angelov S.N., Yan J., Allen J.K., Kang I., Wight T.N. (2015). Postnatal Deletion of the Type II Transforming Growth Factor-beta Receptor in Smooth Muscle Cells Causes Severe Aortopathy in Mice. Arter. Thromb. Vasc. Biol..

[59] Kaur G., Dufour J.M. (2012). Cell lines: Valuable tools or useless artifacts. Spermatogenesis.

[60] Wu X., Wang S., Li M., Li J., Shen J., Zhao Y., Pang J., Wen Q., Chen M., Wei B. (2020). Conditional reprogramming: Next generation cell culture. Acta Pharm. Sin. B.

[61] Bulcha J.T., Wang Y., Ma H., Tai P.W.L., Gao G. (2021). Viral vector platforms within the gene therapy landscape. Signal. Transduct. Target. Ther..

[62] Cante-Barrett K., Mendes R.D., Smits W.K., van Helsdingen-van Wijk Y.M., Pieters R., Meijerink J.P. (2016). Lentiviral gene transfer into human and murine hematopoietic stem cells: Size matters. BMC Res. Notes.

[63] Bi L., Wacker B.K., Stamatikos A., Sethuraman M., Komandur K., Dichek D.A. (2021). Jugular Vein Injection of High-Titer Lentiviral Vectors Does Not Transduce the Aorta-Brief Report. Arter. Thromb. Vasc. Biol..

[64] Stamatikos A., Dronadula N., Ng P., Palmer D., Knight E., Wacker B.K., Tang C., Kim F., Dichek D.A. (2019). ABCA1 Overexpression in Endothelial Cells In Vitro Enhances ApoAI-Mediated Cholesterol Efflux and Decreases Inflammation. Hum. Gene Ther..

[65] Nalbantoglu J., Pari G., Karpati G., Holland P.C. (1999). Expression of the primary coxsackie and adenovirus receptor is downregulated during skeletal muscle maturation and limits the efficacy of adenovirus-mediated gene delivery to muscle cells. Hum. Gene Ther..

[66] Orlicky D.J., Schaack J. (2001). Adenovirus transduction of 3T3-L1 cells. J. Lipid Res..

[67] Arnberg N. (2009). Adenovirus receptors: Implications for tropism, treatment and targeting. Rev. Med. Virol..

[68] Ricobaraza A., Gonzalez-Aparicio M., Mora-Jimenez L., Lumbreras S., Hernandez-Alcoceba R. (2020). High-Capacity Adenoviral Vectors: Expanding the Scope of Gene Therapy. Int. J. Mol. Sci..

[69] Russell R.A., Vassaux G., Martin-Duque P., McClure M.O. (2004). Transient foamy virus vector production by adenovirus vectors. Gene Ther..

[70] Wong C.M., McFall E.R., Burns J.K., Parks R.J. (2013). The role of chromatin in adenoviral vector function. Viruses.

[71] Kim T.K., Eberwine J.H. (2010). Mammalian cell transfection: The present and the future. Anal. Bioanal. Chem..

[72] Chong Z.X., Yeap S.K., Ho W.Y. (2021). Transfection types, methods and strategies: A technical review. PeerJ.

[73] Dyson M.R. (2016). Fundamentals of Expression in Mammalian Cells. Adv. Exp. Med. Biol..

[74] Yang X.P., Amar M.J., Vaisman B., Bocharov A.V., Vishnyakova T.G., Freeman L.A., Kurlander R.J., Patterson A.P., Becker L.C., Remaley A.T. (2013). Scavenger receptor-BI is a receptor for lipoprotein(a). J. Lipid Res..

